# Exploring the Valuable Carotenoids for the Large-Scale Production by Marine Microorganisms

**DOI:** 10.3390/md16060203

**Published:** 2018-06-08

**Authors:** Javier Torregrosa-Crespo, Zaida Montero, Juan Luis Fuentes, Manuel Reig García-Galbis, Inés Garbayo, Carlos Vílchez, Rosa María Martínez-Espinosa

**Affiliations:** 1 Department of Agrochemistry and Biochemistry, Biochemistry and Molecular Biology division, Faculty of Science, University of Alicante, Ap. 99, E-03080 Alicante, Spain; javitorregrosa@ua.es; 2 Algal Biotechnology Group, University of Huelva, CIDERTA and Faculty of Science, Marine International Campus of Excellence (CEIMAR), Parque Huelva Empresarial S/N, 21007 Huelva, Spain; mariazaida.montero@hotmail.com (Z.M.); juanlufc@gmail.com (J.L.F.); garbayo@dqcm.uhu.es (I.G.); bital.uhu@gmail.com (C.V.); 3 Department of Nutrition and Dietetics, Faculty of Health Sciences, University of Atacama, Copayapu 2862, CP 1530000 Copiapó, Chile; manuel.reig@uda.cl

**Keywords:** carotenoids, antioxidants, bioactive compounds, blue biotechnology, marine microorganisms

## Abstract

Carotenoids are among the most abundant natural pigments available in nature. These pigments have received considerable attention because of their biotechnological applications and, more importantly, due to their potential beneficial uses in human healthcare, food processing, pharmaceuticals and cosmetics. These bioactive compounds are in high demand throughout the world; Europe and the USA are the markets where the demand for carotenoids is the highest. The in vitro synthesis of carotenoids has sustained their large-scale production so far. However, the emerging modern standards for a healthy lifestyle and environment-friendly practices have given rise to a search for natural biocompounds as alternatives to synthetic ones. Therefore, nowadays, biomass (vegetables, fruits, yeast and microorganisms) is being used to obtain naturally-available carotenoids with high antioxidant capacity and strong color, on a large scale. This is an alternative to the in vitro synthesis of carotenoids, which is expensive and generates a large number of residues, and the compounds synthesized are sometimes not active biologically. In this context, marine biomass has recently emerged as a natural source for both common and uncommon valuable carotenoids. Besides, the cultivation of marine microorganisms, as well as the downstream processes, which are used to isolate the carotenoids from these microorganisms, offer several advantages over the other approaches that have been explored previously. This review summarizes the general properties of the most-abundant carotenoids produced by marine microorganisms, focusing on the genuine/rare carotenoids that exhibit interesting features useful for potential applications in biotechnology, pharmaceuticals, cosmetics and medicine.

## 1. Introduction

Carotenoids are a class of pigments distributed ubiquitously in nature [1]. These pigments have received considerable attention because of their biotechnological applications and, more importantly, due to their potential beneficial applications in the fields of human healthcare, food processing, pharmaceuticals and cosmetics [2,3]. Chemically, these compounds are mainly C_40_ lipophilic isoprenoids that range from colorless to yellow, orange and red [1,2,4]. These pigments may be categorized into two groups based on the presence or absence of oxygen in their structures: carotenes (do not contain oxygen) and xanthophylls (contain oxygen). The production of these pigments from plants, fungi and certain microorganisms, on a medium-scale level, and even on a large-scale level, has been described previously [5,6,7,8,9,10,11]. In fact, the studies focused on the regulation of carotenogenesis, the biological roles performed by the carotenoids, the general characterization of the carotenoids and the analytical procedures utilized in order to describe their structure are abundantly available in the literature [1,12,13].

Carotenoids have received much attention because of the variety of important biological roles they perform in all living systems [1,14,15,16]. In the majority of organisms, the most relevant biological functions performed by the carotenoids are associated with their antioxidant properties, which are a direct outcome of their molecular structure [10]. Xanthophylls, for instance, perform the role of free-radical scavengers, potent quenchers of ROS (reactive oxygen species) and RNS (reactive nitrogen species) and chain-breaking antioxidants. Therefore, astaxanthin and canthaxanthin (which are xanthophylls) are better antioxidants and scavengers of free radicals compared to β-carotene. In recent years, understanding the ROS-induced oxidative stress mechanisms and the search for suitable strategies in order to fight oxidative stress have become the major goals of medical research efforts [10]. Furthermore, carotenoids are the natural compounds that are responsible for conferring color to animals, plants and microorganisms [17].

Animals, as well as humans are not capable of synthesizing the carotenoids de novo; the pigments are, therefore, acquired through diet*.* However, they are capable of converting these pigments into vitamin A and the retinoid compounds, which are required for morphogenesis and embryonic development [18,19]. Vitamin A is a well-recognized factor of great importance in child health and survival; its deficiency causes disturbances in vision and also lung, trachea and oral cavity pathologies. Consuming carotenoids through diet is the only way to carry out retinol synthesis in animals and humans; fruits, vegetables and microalgae being the major sources of carotenoids that exhibit pro-vitamin A activity [10,19]. Other biological roles and functions performed by the carotenoids in animals and humans include absorption of light energy, oxygen transport [20], enhancing in vitro antibody production and antitumor activity [21,22] and antioxidant and anti-inflammatory activities [23].

In birds and fishes, carotenoids are an important indication of a satisfactory nutritional condition and are used in ornamental displays as a sign of fitness and to increase sexual attractiveness [16,24,25]. In algae and higher plants, carotenoids serve as the regulators of plant growth and development, the accessory pigments for photosynthesis and as photoprotectors. Thus, they contribute to light harvesting, maintenance of the structures and functions of the photosynthetic complexes, quenching of the chlorophyll triplet states, scavenging of reactive oxygen species and dissipation of excess energy [26]. Besides, carotenoids also serve as precursors for certain hormones such as abscisic acid (ABA) and strigolactones, as well as attractants for other organisms, such as insect pollinators and seed-dispersing herbivores [19]. Apart from the above-mentioned important roles, carotenoids also serve as important floral pigments, due to their striking and rich color, to attract pollinators and seed dispersers. Microorganisms are a great source of a variety of carotenoids. In microorganisms, carotenoids oversee photoprotection, provide color to microbial cells and regulate the mechanisms against oxidative stress. It is important to highlight that certain carotenoids, such as salinixanthin or bacterioruberin (produced by halophiles), are produced solely by certain extremophilic microorganisms [8,27,28].

For the last 30 years, researchers and the R&D companies have paid much attention to the microorganisms that are capable of producing significant concentrations of carotenoids, because these biocompounds obtained from these natural sources are in high demand these days. This huge demand is a result of consumers’ preference for natural as opposed to synthetic products. Such demand has encouraged the production of scientific literature on novel carotenoid-producing microorganisms and has prompted major efforts to enhance the isolation of carotenoids from their biological sources instead of their chemical synthesis. This fact, coupled with fresh insights into the molecular biology techniques and downstream processes, epitomizes the carotenoid-producing microorganisms as suitable natural sources for the large-scale synthesis of carotenoids, as an alternative to in vitro carotenoid production. Among all the natural sources of carotenoids, marine microorganisms have emerged as the natural sources from which the production of these pigments may be relatively easy.

This review is an attempt to compile the latest studies and research works in this field. It summarizes the main findings that have been described to date about the marine microbial carotenoids, highlighting their potential beneficial effects on human health and their relevance in the natural compounds market.

## 2. Marine Microorganisms as a Source of Bioactive Compounds

Bioactive compounds are the compounds that produce specific effects within a living organism, tissue or cell. In the field of nutrition, bioactive compounds have been distinguished from essential nutrients; essential nutrients are necessary for the sustainability of life, while bioactive compounds are not. However, bioactive compounds may have an influence (usually positive) on metabolism and on health in general. Bioactive compounds are usually the secondary metabolites produced by microorganisms, yeast, algae and fungi [29].

Marine life forms have long adapted their physiology and metabolism to the extreme ambient conditions present in the seas, oceans and other closely-related environments, such as marshes, coastal salt ponds, etc. Consequently, they have evolved within themselves protective mechanisms that include the accumulation of bioactive compounds [30,31]. Recent research on marine ecosystems has revealed that the marine aquatic biomass (free-living cells or symbiotic) and its bioactive compounds have many potential applications in various fields; for example, ensuring future food and nutrition security and supplying raw materials for other high added-value chains and products, such as bioenergy, pharmaceuticals and cosmetics, while factoring in the environmental and climate change risks.

Therefore, international institutions, such as the European Commission, are promoting investigations in the field of biotechnology in general, particularly in blue biotechnology, a field that is concerned with the exploration and exploitation of the resulting diversity of marine organisms in order to develop novel products [32]. Besides, a significant number of research groups are working in a coordinated manner to improve the knowledge about carotenoids and the novel natural sources for their production, as is evident from the presence of networks such as Eurocaroten [33], CaRed (the Spanish Carotenoid Network; [34], the International Carotenoid Society [35] and IBERCAROT (the Ibero-American network for the study of carotenoids as functional food ingredients, [36]).

In line with this fact, the microorganisms that produce a significant amount of bioactive compounds (lipids, carotenoids, vitamins, etc.) are abundant in the marine environment; therefore, they are considered suitable targets for exploring their potential as the natural biosources from which carotenoids may be obtained at a large scale [37,38,39]. Besides, modern approaches such as genomics, transcriptomics, proteomics, etc., have been recently used as powerful tools to investigate the production of bioactive compounds from marine organisms [40,41,42]. Therefore, marine plants (kelp, for instance), phytoplanktons, marine algae and microorganisms such as marine bacteria and Haloarchaea, are perceived as attractive sources for the production and isolation of common, as well as novel and rare carotenoids (Table 1).

Marine microorganisms, in addition to their capacity to synthesize unique bioactive compounds, offer certain advantages specific to a large-scale production of carotenoids; for example, the risk of contamination with other microorganisms is reduced due to the high-salinity conditions used in their culture media [43]. This feature becomes more significant when the extreme halophilic marine microorganisms such as Haloarchaea are used as the natural source of bioactive compounds [8,44]. However, the concern is to produce the bioactive compounds at a price that is competitive; which is difficult because the production costs of the microbial biomass are high. Therefore, further investigation is required to achieve a more profitable production of the bioactive compounds.

## 3. Properties of the Most Demanded Carotenoids Isolated from Marine Microorganisms

This section summarizes the general aspects of the carotenoids that are most demanded in the pharmaceutical, cosmetics and biotechnological markets. These are known as the common carotenoids. These carotenoids are present in marine microorganisms, as well as in other organisms, such as terrestrial plants, aquatic plants, fungi, yeast, etc. Although these carotenoids have been obtained mainly from plants, fungi, yeast and algae, several recent works on microorganisms (mainly from the marine environments) have revealed that certain microbial genera are the producers of significantly large amounts of several carotenoids with potential applications in biomedicine and biotechnology.

Table 2 enlists the main features of the structure of each of the following carotenoids.

### 3.1. Astaxanthin

Astaxanthin (3,3′-dihydroxy-β,β’-carotene-4,4′-dione) is a red-pink-colored xanthophyll carotenoid, which contains two additional oxygenated groups on each ring compared to the other carotenoids, which results in enhanced antioxidant properties of this compound [56]. It is a β-carotene derivative, which is eleven-times more potent as a quencher of singlet oxygen than β-carotene and 550-times more potent than α-tocopherol [54]. Astaxanthin exhibits a higher biological activity than the other antioxidants, because of its ability to link across the entire cell membrane from the inside to the outside [87]. This compound occurs naturally in a wide variety of living organisms, including microalgae, fungi, plants, marine organisms and certain birds such as flamingos; it confers salmon, shrimp and lobster their distinctive color [54]. Astaxanthin can neither be synthesized de novo by animals, nor converted into vitamin A; therefore, it must be consumed in the diet [56]. The high potency, as well as the polar property of astaxanthin makes it an attractive antioxidant nutraceutical suitable for further investigation in the field of biomedicine. Several works have demonstrated that astaxanthin, when used as a nutritional supplement (it is associated with the E number E161j), has potential beneficial effects on human health. The biotechnological production of astaxanthin from various sources has been studied in depth [88] in order to achieve its production on a large scale for several commercial applications [87]. The astaxanthin products are used for commercial applications in dosage forms, such as tablets, capsules, syrups, oils, soft gels, creams, biomass and granulated powders. Astaxanthin-related patent applications are available in the areas of food, feed and nutraceuticals and are currently the major market driver for the pigment.

### 3.2. β-Carotene

β-Carotene is an intensely-colored orange pigment, abundant in green leafy plants (parsley, spinach, broccoli), certain fruits (mandarin, peach) and several vegetables (carrot, pumpkin) [26]. Its distinguishing characteristic is the beta rings present at both ends of the molecule. β-Carotene occurs as several isomers, two of which (9-*cis* and all-*trans*) constitute approximately 80% of the total β-carotene present in the microalga *Dunaliella bardawil* [89]. β-Carotene is used as a food coloring agent with the E number E160 [90]. In nature, β-carotene is a precursor (inactive form) of vitamin A, which is synthesized from carotenoids via the action of the enzyme β-carotene 15,15′-monooxygenase. Following its synthesis, vitamin A is assimilated or further converted into retinoids so that it does not cause hypervitaminosis A. The isolation of β-carotene from fruits is commonly performed by using column chromatography [91,92]. β-Carotene is deeply colored and a highly-conjugated carotenoid, which lacks functional groups, causing it to be highly lipophilic. Overconsumption of β-carotene may cause carotenosis, a benign condition under which the skin turns orange. Chronic intake of high doses of β-carotene supplementation has been correlated directly with the increase in the probability of lung cancer in cigarette smokers [93].

### 3.3. Canthaxanthin

Canthaxanthin (β,β-carotene-4,4′-dione) is a diketo carotenoid pigment, which is orange-red in color. It was first isolated from edible mushrooms. In several green algae and also in blue-green algae, this pigment is produced as a secondary carotenoid at the end of the growth phase, either in place of, or in addition to, the primary carotenoids. It has also been found in bacteria, crustaceans, birds (even in the yolk of bird eggs) [94] and in various species of fish, including the common carp (*Cyprinus carpio*), golden mullet (*Mugil auratus*), annular seabream (*Diplodus annularis*) and thrush wrasse (*Crenilabrus tinca*) [57]. It is used as a food coloring agent, associated with the E number E161g, in different countries, including the United States and the EU member states. As canthaxanthin has a high commercial value, its biosynthesis has been studied extensively. Canthaxanthin is biosynthesized from β-carotene, through the action of a single enzyme, known as β-carotene ketolase, which adds carbonyl groups to the carbon atoms at the 4 and 4′ positions in the β-carotene molecule. Although functionally identical, several distinct β-carotene ketolase proteins are known, which differ (from an evolutionary perspective) in their primary protein/amino acid sequence. In order to improve the large-scale canthaxanthin production using microorganisms, the regulation of its biosynthesis has been studied recently in *Haematococcus pluvialis* [95] and in *Dietzia natronolimnaea* [96,97].

### 3.4. β-Cryptoxanthin

β-Cryptoxanthin (hydroxy-β-carotene) occurs in a variety of sources, including petals of flowers and several fruits such as papayas, satsuma mandarins and apples [57]. It is present together with α-carotene, β-carotene, lycopene, lutein and zeaxanthin. It is also present in egg yolk [98] and sweet oranges [99]. β-Cryptoxanthin is a good source of vitamin A, and therefore, it is considered a pro-vitamin A [100]. This carotenoid is oxidized and isomerized in the presence of light [101]. β-Cryptoxanthin is used as a coloring agent to color food products in certain countries and is associated with the E number E161c. β-Cryptoxanthin obtained from its common food sources, exhibits relatively high bioavailability to the extent that certain β-cryptoxanthin-rich foods might be considered equivalent to β-carotene-rich foods as sources of retinol [102].

### 3.5. Fucoxanthin

Fucoxanthin is one of the most abundant carotenoids, and it is present as an accessory pigment in the chloroplasts of brown algae, phytoplankton, brown seaweeds and diatoms, giving them a brown or olive-green color [58]. It shares more than 10% of the estimated total production of carotenoids in nature, especially in the marine environments [57]. Fucoxanthin is a xanthophyll with a unique structure that includes an unusual allenic bond, an epoxide group and a conjugated carbonyl group in the polyene chain, conferring it the antioxidant properties. However, the difference is that fucoxanthin exhibits antioxidant properties even under anoxic conditions, while the other carotenoids demonstrate practically no quenching ability under those conditions. Most of the tissues have a low-oxygen status under physiological conditions. Consequently, fucoxanthin may be performing key roles under anoxic conditions. The unique molecular structure of fucoxanthin confers remarkable biological properties to it, similar to neoxanthin, dinoxanthin and peridinin. Fucoxanthin does not exhibit toxicity and mutagenicity under experimental conditions, and it may possess the ability to increase the levels of circulating cholesterol in rodents as a common feature [57]. Fucoxanthinol is the deacetylated derivative of fucoxanthin. In fact, it has been reported that several of the bioactive properties of fucoxanthin are due to fucoxanthinol [58,103,104,105,106]. Fucoxanthinol exhibits suppressive effects on lipid accumulation during adipocyte differentiation, and it also demonstrates anti-inflammatory and antioxidative properties.

### 3.6. Lycopene

Lycopene (ψ,ψ-carotene) is responsible for the red color in several fruits and vegetables, such as tomatoes. Unlike certain other carotenoids, lycopene lacks the terminal β-ionone ring in its structure, and therefore, provitamin A activity is not present in this carotenoid. Lycopene has a highly unsaturated, hydrocarbon chain, consisting of eleven conjugated and two unconjugated double bonds, which confers its antioxidant activity. Because of the presence of the double bonds in the structure of lycopene, it can exist in both *cis* and *trans* isomeric forms. In nature, lycopene is present primarily in the all-*trans* isomeric form. However, it may undergo mono- or poly-isomerization in the presence of light, thermal energy and temperature, which can convert it to the *cis* isomer. Lycopene is highly stable under the conditions of thermal processing and storage. It has been reported that 5-*cis* lycopene is the most stable isomer of lycopene, followed by the all-*trans*, 9-*cis*, 13-*cis*, 15-*cis*, 7-*cis* and 11-*cis* isomeric forms. The 5-*cis* isomer of lycopene has been demonstrated to exhibit the highest antioxidant activity, followed by the 9-*cis*, 7-*cis*, 13-*cis*, 11-*cis* and all-*trans* isomers [85]. Lycopene is associated with the E number E160d when used as a coloring agent for food products.

### 3.7. Lutein

Lutein (β,ε-carotene-3,3′-diol) is one of the two major components of the macular pigment of the retina and is present at a high concentration in the human serum [107]. It is synthesized only by plants, and similar to the other xanthophylls, it is present in high quantities in green leafy vegetables, such as spinach and kale, and yellow carrot. In green plants, lutein modulates light energy and serves as a non-photochemical quenching agent to deal with triplet chlorophyll (an excited form of chlorophyll), which is overproduced at high light intensity during photosynthesis. Lutein contains only 10 conjugated double bonds, which causes it more yellowish-green color compared to the other carotenoids. Lutein acts as a powerful antioxidant and is able to filter high-energy blue light [108]. When used as a food colorant, it is associated with the E number E161b. Recently, novel methodologies have been developed and optimized in order to isolate this pigment from vegetables, for example from spinach [109] or carrot [92].

### 3.8. Zeaxanthin

Zeaxanthin (β,β-carotene-3,3′-diol) is one of the most common carotenoid alcohols present in nature, which performs an important role in the xanthophyll cycle. It is synthesized by plants and certain microorganisms. Zeaxanthin is the pigment that confers paprika (produced from bell peppers), corn, saffron, wolfberries and several other plants and microorganisms (such as marine bacteria) their characteristic color [110]. It is also one of the two major components of the macular pigment of the retina. Zeaxanthin is isomeric with lutein, and the two carotenoids differ from each other only in the location of a single double bond; in zeaxanthin, all the double bonds are conjugated. Zeaxanthin does not exhibit vitamin A activity. This pigment and its close relative lutein perform a critical role in the prevention of AMD (age-related macular degeneration), one of the leading causes of blindness [57]. Like lutein, zeaxanthin has been found in significant concentrations in human milk [111]. Because of its high value and demand in the nutraceutical market, several methods have been developed to produce zeaxanthin on a large scale [112]. As a food colorant, it is associated with the E number E161 h.

### 3.9. Violaxanthin

Violaxanthin (5,6:5′,6′-diepoxy-5,5′,6,6′-tetrahydro-β-carotene-3,3′-diol) is a natural xanthophyll pigment, which is orange in color. It is present in a variety of brown algae and in plants including pansies. This pigment is biosynthesized from zeaxanthin through the process of epoxidation. As a food additive, it is used under the E number E161e; however, this use is approved only in Australia and New Zealand, where it is listed under the INS number 161e. The interconversions of violaxanthin and zeaxanthin are caused mainly by the action of the violaxanthin de-epoxidase enzyme. Violaxanthin de-epoxidation and the violaxanthin cycle were first studied in the late 1960s to the early 1970s [113,114,115]. Violaxanthin de-epoxidases are susceptible to DTT [116,117], and their thermostability is due to the disulfide bridges present in their structures [118]. These interconversion mechanisms have been observed to correlate directly to the dissipation of excess excitation energy in leaves in 2% O_2_, 0% CO_2_ [119]. So far, the major biological roles of violaxanthin, as well as its role as a precursor of abscisic acid, have been described using leaves [120] and fruits [121] as the sources of this carotenoid.

## 4. The Rare Carotenoids

The rare carotenoids include certain carotenoids recently found in only a few marine organisms, at the time of writing this review. Although there are probably several rare carotenoids to be described, only a few of them have been reported so far in the previous studies. The production of the biomass of marine organisms that contain the rare carotenoids is the first step toward the biotechnological production of these carotenoids. All the rare carotenoids that have been described so far were isolated mainly from microalgae or Haloarchaea (the halophilic microorganisms belonging to domain Archaea, which inhabit salty environments). So far, there are no examples of a large-scale production of rare carotenoids from microalgae, and only one indexed paper has reported the cultivation of *Nostoc* for this purpose [122]. Several wild-types, as well as genetically-engineered species of *Nostoc*, have been used to investigate the production of exopolysaccharides, such as polyhydroxyalkanoates [123], and the production of hydrogen [124]. Moreover, several species of *Anabaena* have also been reported to serve as attractive sources for the production of exopolysaccharides [125] and carotenoids as feedstock for biodiesel [126]. However, the production of rare carotenoids from *Nostoc* and *Anabaena*, as well as the processes for the large-scale production of their biomass have not yet developed completely.

Other rare carotenoids, such as bacterioruberin (and its derivatives) or salinixanthin, are produced mostly from Haloarchaea [8]. The recent works reported by Shindo and by Gamone and co-workers have referred to myxol, saproxanthin, sioxanthin and siphonaxanthin as rare carotenoids [15,127]. The main features describing each of the above-mentioned rare carotenoids are summarized in the following sections, as well as in Table 3.

### 4.1. Bacterioruberin

Bacterioruberin is the main carotenoid responsible for the color of the red halophilic Archaea belonging to the families Halobacteriaceae and Haloferacaceae. To date, the production of this rare carotenoid has been reported only from the Haloarchaea and *Micrococcus roseus* [8,128]. Bacterioruberin is a C_50_ carotenoid pigment, exhibiting a unique molecular structure. It consists of a primary conjugated isoprenoid chain with 13 C=C units and no subsidiary conjugation arising from the terminal groups, which contain only four hydroxyl groups [8]. This pigment protects the microbial cells against the damage caused by high intensities of light in the visible and the ultraviolet range of the spectrum, and it also aids in photoreactivation [129]. The pigment is also involved in the reinforcement of the microbial cell membrane. This pigment was first described from the cells of a *Halobacterium* species [130]. Recent works on the production of bacterioruberin from Haloarchaea report that the production of this rare carotenoid may be easily enhanced by modifications of the temperature, salt concentrations, pH, oxygen availability or light incidence [8,44].

### 4.2. Myxol

Myxol is derived from γ-carotene. It is present in various forms in nature; however, as free myxol, it is present mainly in the marine environments. The marine bacterium MBIC 03313 was reported as the first microorganism to produce free myxol. *Anabaena variabilis* ATCC 29413 also produces this pigment, along with 4-hydroxymyxol [131]. *Robiginitalea myxolifaciens* sp. nov. has been reported to be capable of producing myxol in sea water [132]. Myxol, in its glyoxylated form, has been identified in both marine and non-marine aquatic microorganisms such as *Nostoc commune* [133]. The freshwater alga *Oscillatoria limosa* produces myxol-glyoxylate as myxol-2′-O-methyl-methylpentoside and 4-keto-myxol-2′-methylpentoside [73]. The potential applications of myxol in the area of human health are related to its strong antioxidant activity. Myxol and saproxanthin have demonstrated better antioxidant activity against lipid peroxidation and better neuroprotective effects against L-glutamate toxicity than β-carotene or zeaxanthin, in a rat brain homogenate model [134]. The antioxidant activity of myxol has also been associated with its capacity to decrease the oxygen permeability in lipid membranes [15].

### 4.3. Salinixanthin

This carotenoid is produced mainly by the halophilic bacterium Salinibacter ruber and by certain halophilic Archaea species belonging to the Halobacteriaceae and Haloferacaceae families [8,28,135]. The structure of salinixanthin ((all-E,2′S)-2′-hydroxy-1′-[6-O-(13-methyltetradecanoyl)-β-D-glycopyranosyloxy]-3′,4′-didehydro-1′,2′-dihydro-β,Ψ-caroten-4-one) was first determined using spectroscopic techniques [136]. In nature, this carotenoid functions as a light-harvesting antenna for supplying the additional excitation energy for retinal isomerization and proton transport [137].

### 4.4. Saproxanthin

Saproxanthin is a xanthophyll carotenoid. It was first reported and described by Aasen and Jensen in Saprospira grandis. A selected strain of Saprospira sp., SS90–1, which was identified as Saprospira toviformis, synthesizes saproxanthin as a major pigment [138]. The marine bacterial strain 04OKA–13–27 was reported as the second species to produce saproxanthin [127,134]. More recently, it was reported that alkaline conditions improved the synthesis of this rare carotenoid in the bacterial strains belonging to Jejuia pallidilutea [139].

### 4.5. Sioxanthin

Sioxanthin has been reported to be produced so far only by a marine actinomycete belonging to the genus Salinispora. It was described that this pigment has a novel C40 carotenoid structure, which is glycosylated at one end of the molecule, and contains an aryl moiety at the other end. Glycosylation is unusual among the actinomycete carotenoids, and therefore, sioxanthin forms a part of the rare group of compounds that possess polar, as well as non-polar head groups [140].

### 4.6. Siphonaxanthin

Siphonaxanthin is a keto-carotenoid, a xanthophyll pigment that is present in the species belonging to the Siphonales order [15,141]. Siphonales are the green algae present in both marine and freshwater environments and are quite common in deep, as well as shallow waters [142,143]. To date, the species that belong to this order are the only ones identified as the producers of siphonaxanthin. The characterization of this rare carotenoid remains poorly explored; nevertheless, several recent works have stated that siphonaxanthin could be considered a bioactive compound with potential beneficial effects on human health [15,144].

## 5. Carotenoids and Human Health

To date, more than 6000 works on the effects of carotenoids on human health have been published in the indexed scientific journals (PubMed: 6500; Scopus 3000; and WOS: 5978; “carotenoids” and “human health” were used as keywords to perform the search). Most of the studies have reported the general characterizations of carotenoids using biochemical techniques and the conclusions regarding their potential benefits [17]. In certain other works, the potential effects of carotenoids on cell lines or animal models have been described. Basically, only one-third of the research currently published in this area of study has been conducted in humans. Therefore, so far, the studies demonstrating a direct correlation between the carotenoid uptake and the beneficial effects on human health are scarce. Another important aspect that needs to be highlighted here is that the assays that are used to analyze the carotenoids in the human body must be optimized, as several of the studies continue to use empirical prediction models instead of real-time measurements in human fluids or tissues [145]. Consequently, further research is required in both basic and applied aspects.

The few connections that have been established directly between carotenoid consumption and the human health reveal that: (i) these pigments are powerful antioxidants, and consequently, they could possess antitumor activity; (ii) certain carotenoids exhibit pro-vitamin A activity; and (iii) certain carotenoids may be able to demonstrate regulatory activities at different levels in several tissues, and because of this regulatory role, they may exhibit a protective effect against the development of degenerative diseases, such as macular degeneration, cancer and heart diseases [15], or they may be able to prevent metabolic diseases, such as type 2 diabetes or dyslipidemia [146,147]. Table 1 summarizes the major potential beneficial effects of carotenoids (or their derivatives) on human health, as described by various studies, to date.

Nowadays, consumers are aware of the association among diet, health and the prevention of diseases [147]. As a result, bioactive compounds, such as antioxidants, peptides, carbohydrates and lipids, present in food have become important for human nutrition and the reason for the development of functional foods and nutraceuticals in the food industry. The terms such as “health-promoting foods” or “functional foods” refer to food that is rich in bioactive compounds and substances that are effective against diseases [148]. The outer appearance of these functional foods is similar to conventional foods; however, the difference is that functional foods offer advantages beyond the basic nutritional functions; they exhibit physiological benefits and are able to reduce the risk of chronic diseases [149]. The term “nutraceutical” was coined by combining two terms, “nutrition” and “pharmaceutical”, in 1989 [150]; nutraceuticals are defined as “a food (or a part of food) that provides medical or health benefits, including the prevention and/or treatment of a disease” [150]. When the functional food aids in the prevention and/or treatment of a disease(s) and/or a disorder(s) other than anemia, it is referred to as a “nutraceutical” [151]. It is important to note that functional foods may not be a universal panacea for poor health habits [152]. In brief, health benefits and novel markets in the food industry may be obtained by incorporating carotenoids into foods that do not contain high amounts of these natural pigments; also, when added to food, the bioavailability of carotenoids is improved compared to when they are consumed from their natural sources [153].

The interest in marine-derived functional foods has increased, especially the functional foods from marine algae, which are considered an interesting source of bioactive compounds with biological properties to be used as functional food ingredients. Taking into consideration all of these aspects, carotenoid-rich foods, such as the Mediterranean diet, are being regarded as the best diet models for supporting healthy living standards and to promote the positive effects on human health, for instance cardiovascular health [15]. Host-related factors may also affect the capability to absorb, convert and metabolize dietary carotenoids. Factors such as gender, body fat and genetic variation may also play important roles in these processes.

It is noteworthy that several recent works have stated that the intake of food supplements without professional supervision is associated with certain problems, such as side effects caused when the supplements interact with classical synthetic drugs [154]. The excessive intake of carotenoids causes carotenemia in humans, which is benign. Moreover, unusual diets and the consumption of preformed vitamin A present in the food supplements may lead to vitamin A toxicity [155,156]. In summary, a significant number of previous studies support the use of food supplements, nutraceuticals and carotenoid-rich foods; however, in all cases, the consumption of these supplements must be accompanied by professional/medical supervision.

## 6. Marine Microalgae Biomass and Their Valuable Molecules in the Food Market

Microalgae have been used as food, feed and fertilizers for centuries. Their cultivation on a large scale may be extended to areas that are unsuitable for agricultural purposes, with productivities higher than those obtained with traditional crops [9]. Marine microalgae are rich in PUFAs (poly-unsaturated fatty acids), polysaccharides, sterols and pigments such as carotenoids, chlorophylls and phycobiliproteins [157,158] that could be used to increase the nutritional value of foods [159]. Antioxidants have become a major focus of interest, and several studies have associated antioxidant activity with carotenoid content in algae [160], which has resulted in an increased demand for algae in order to obtain carotenoids to be added to functional foods.

The global market for carotenoids is expected to reach U.S. $1.7 billion by the year 2022. The market size of functional foods derived from microalgae has increased five-fold since the beginning of the century, and its development has relatively matured now [161]. Microalgae are currently used both as dried whole algae and as the source for the extraction of high-valued food supplements, such as carotenoids and omega-3 fatty acids. A majority of microalgae used belong to the genera *Chlorella*, *Dunaliella*, *Spirulina*, *Haematococcus*, *Isochrysis* and *Schizochytrium* [162]. *Chlorella* and *Spirulina* are the most-marketed algae worldwide. The production of *Chlorella* is centered in Asia, while *Spirulina* is produced in Asia, as well as the USA; although both of these microalgae are also produced in a few other countries with warm climates [163]. Both *Chlorella* and *Spirulina* rank first in the microalgal biomass production rate worldwide (5000 and 2000 tons of dry weight/year, respectively), with estimated production values of about $40 million/year for each [164]. In food, carotenoids serve as the precursors to aroma compounds and also as natural antioxidants that may help prolong the shelf life of food products [165]. Therefore, Portugal [9,166,167] proposed to utilize microalgal biomass as pigments and functional ingredients in food products. The biomass of *C. vulgaris* and *H. pluvialis* has been incorporated into pea protein-stabilized emulsions, achieving a considerable compromise between the stability and the sensory properties. The *C. vulgaris* biomass, with organoleptic characteristics that are acceptable to consumers, was used as a source of natural green pigment to color Christmas cookies.

A fishy taste and a powdery consistency are a few inconveniences caused by the incorporation of microalgal biomass in traditional food products. It may be necessary to address the issues concerning these organoleptic characteristics, for the food products to be accepted by many consumers [147]. In the traditional cuisines of Asian countries, algae have been a common ingredient. As a result, the addition of microalgae to food is not perceived as a strong change and is appreciated by the consumers in these countries [168]. In Western nations, however, microalgae are considered a novel ingredient, and their addition to food has not been accepted yet.

Instead of using the whole biomass, Gantar and Svircev [169] suggested employing the microalgal biomass as a source of biomolecules of interest, so that it would be accepted by consumers [162]. Microalgae-based high-valued molecules have been produced on a smaller scale, with a larger market potential, mainly in Asia, the USA and Australia. Although the production costs are high, the quality of biomolecules produced is better than that from the alternative methods, such as chemical synthesis or the extraction from plants; this is mainly because the molecules produced from the microalgal biomass is more effective for food applications compared to their synthetic variants [170]. The most important molecules from the microalgal biomass that are currently on the market are carotenoids and fatty acids used as dietary supplements. Astaxanthin from dried *H*. *pluvialis*, which is used as a food additive or as a dietary supplement, is the most-developed product from this source [171].

As a result of the development of novel eating habits among consumers who are now demanding more sophisticated products, a few innovative food products that are enriched with microalgae have been developed and positioned in this emerging functional food market. One good example is *Spirulina*, an ingredient in beverages and shakes [162], which is used in combination with other ingredients. It is available in New Zealand as Charlie’s Honest Superfood Spirulina and Fruit Smoothie. Coca-Cola’s brand Odwalla manufactures a fruit juice drink with *Spirulina* as an ingredient [162]. Another example is PepsiCo, 100% Juice Smoothie with *Spirulina* [172]. Nestle Rowntree reintroduced the blue Smarties into the market after having identified the blue-green cyanobacteria as a natural source of the blue color [173].

Besides the health interests, it is important to assess the toxicity of the natural compounds isolated from microalgae (pigments, lipids, etc.), as certain compounds may accumulate in the human body, and this could affect the safe usage of microalgae in food products for human consumption [174]. It is undeniable that the interest in using microalgae or the other marine microorganisms as natural sources for functional food ingredients is growing, and certain reasons that support this fact are as follows: the number of marine species available and the prospect of discovering novel ones is significant [175], and the management of growth conditions is able to assist the marine microorganisms in accumulating bioactive compounds, which is helpful in economically-competitive processes [8,44,160].

### Regulation

The regulations for the production of marine microbial biomass (or their bioactive compounds) are poorly established, increasing the chaos worldwide in this regard. Besides, the laws and regulations associated with food or food ingredients vary with different countries [176]. There are two EU regulations for the production and marketing of microalgae-based products for food and feed: the Food Safety Regulation (EC 178/2002) and the Novel Food Regulation (EC 258/97) (MB). The latter is particularly relevant, as it provides the authorization procedures for all novel food and feed products. In Europe, the first step to commercialize carotenoids or the microorganisms as food or food ingredients is to identify whether they have been consumed to a significant degree before 15 May 1997 (the date on which the regulation (EC) No. 258/97 entered into force), in at least one of the member states. If this is the case, the food or the food ingredient should be considered the same as a conventional food, if it demonstrates the same characteristics and composition as those of the conventional food. This simplifies the http://www.linguee.es/ingles-espanol/traduccion/commercialization+process.html commercialization process, and the food product may be positioned in the market within the EU after notification to the European Commission [177]. Prior to commercializing any type of novel food product (including the ones from microalgae), scientific evidence must be provided in order to confirm that the novel food products are equivalent to the traditional ones (EC Regulation, 1997) [178].

In another scenario, if the food or the food ingredient is identified as a novel food, the further procedure becomes complex. In order to introduce a novel food or food ingredient into the EU market, prior authorization is required, which includes the manufacturing processes, as well as a rigorous assessment of the toxicological, nutritional, compositional and other relevant data by the competent authorities of the respective EU member state. In the event of commercialization of the microorganism as a functional food, it must be demonstrated to affect one or more target functions in the body beneficially. The functional foods are not allowed to be in the form of pills or capsules; they must remain in the form of food. Furthermore, they must be demonstrated to achieve their effects when used in an amount that would normally be expected to be consumed in the diet (EAS 2008) [164].

Regulations in the USA assess the food product, while those in Europe are focused on the technology used to obtain the final food product. The competition from outside Europe (China and the USA) is growing fast, and this is the right time for Europe to explore the opportunities to increase the possibility of successful large-scale applications of microalgae and other marine microorganisms in food [149]. The insufficient domestic demand for marine microalgal food products in Europe and the difficulties in achieving commercial authorization due to the strict Novel Food regulation remain a challenge. In this regard, experts consider cost reductions, technical breakthroughs and achieving better cooperation between academia and industries as the most important challenges in the near future [164].

## 7. Conclusions

In the last few decades, the research on carotenoids, and their implementation in markets, have advanced considerably. However, further advancement of this development is required, especially in the processes used to obtain carotenoids; for example, chemical synthesis must be replaced by biological production. In this way, the reduction in the costs of the production of carotenoids and in the generation of non-active waste would be achieved.

Marine microorganisms offer numerous advantages as natural sources of carotenoids: they usually have low nutritional requirements during their growth and cultivation, which reduces the production costs; the culture medium in which they grow contains moderate or high salt concentrations, which prevents contamination with other microorganisms, reducing the costs and facilitating the downstream processes; these microorganisms are also a source of rare carotenoids, the properties of which are yet to be explored, for example bacterioruberin from Haloarchaea.

In a more applied sense, it should be noted that an extensive bibliography is available on the biochemical characteristics of carotenoids and their potential beneficial effects on human health. However, further studies on the direct and real-time effects of carotenoids on human populations are required, in order to corroborate their antioxidant, antitumor, anti-aging and various other roles. On the nutritional level, the use of the carotenoids obtained from marine biomass is well studied; also, their use is common in the diets of people in several countries, especially in Asia. The industry must continue to take steps in the direction of improving the organoleptic properties, which are altered by the addition of marine biomass to the conventional foods; especially in Europe, where such functional foods are relatively recent and not yet completely accepted.

## Figures and Tables

**Table 1:** Microorganisms that produce carotenoids in the marine environment and biological properties with potential benefits for human health.

Abundant Carotenoids in the Marine Environment			
Marine Microorganism	Carotenoid	Biological Properties	References
Microalgae Bacteria Cyanobacteria	β-Carotene	Antioxidant immune response Anti-inflammatory Benefits for cognitive function and atherogenesis Antidiabetic activity Antitumor activity	[10,45,46,47]
Haloarchaea Bacteria Cyanobacteria Microalgae	Astaxanthin	Immune response anti-inflammatory Antioxidant activity Antitumor activity Ocular protective effect Antidiabetic activity Against: ○ benign prostatic hyperplasia ○ cancer ○ asthma ○ rheumatoid arthritis ○ metabolic syndrome ○ diabetic nephropathy ○ cardiovascular diseases ○ neurodegenerative diseases	[8,48,49,50,51,52,53,54,55]
Microalgae	Fucoxanthin	Reduction of cardiovascular risk factors Electron donor Involved in lipid metabolism increasing production of energy Antioxidant activity Anti-inflammatory effect Anticancer activity Anti-obese effect Antidiabetic activity Hepatoprotective effect Skin-Protective effect Antiangiogenic effect Cerebrovascular protective effect Bone-protective effect Ocular protective effect Antimalarial effect	[15,51,56,57,58,59,60]
Microalgae Bacteria Cyanobacteria	Zeaxanthin	Reduction of cardiovascular risk factors Prevention of coronary syndromes Helps in maintaining visual function Antitumor activity (breast cancer) Anti-cardiovascular diseases Antioxidative, anti-inflammatory and structural actions in neural tissue	[15,61,62,63,64,65]
Cyanobacteria	β-Cryptoxanthin	Antioxidant Immune response anti-inflammatory Improves respiratory function and lowers lung cancer rates Stimulation of bone formation Reduces the rate of oral and pharyngeal cancer Modulation response to phytosterols in post-menopausal women Protection of leukocyte telomeres’ length Decreases risk of some cancers and degenerative diseases. Bone-protective effect	[15,66,67,68,69,70,71]
**Less abundant carotenoids in the marine environment**			
Haloarchaea	Bacterioruberin	Antioxidant Anticancer activity	[8]
Bacteria	Saproxanthin	Antioxidant Apoptosis-inducing effect	[15,72]
Cyanobacteria Bacteria	Myxol	Antioxidant Anticancer activity Against cardiovascular pathologies	[15,73]
Actinomycetes	Sioxanthin	Antioxidant	[74]
Microalgae Haloarchaea	Lutein	Antioxidant Anti-macular eyes degradation Prevention coronary syndromes and stroke Prevention of cataract Prevention of retinitis Ocular protective effect Dopaminergic neurons protection against MPTP-induced apoptotic death Anti-atherosclerosis	[8,15,62,74,75,76,77]
Microalgae Cyanobacteria Haloarchaea Bacteria	Canthaxanthin	Antioxidant Antitumoral activity Provitamin A activity	[8,46,51,78,79,80]
Microalgae Cyanobacteria	Echinenone	Antioxidant	[51,74,81]
Microalgae	Violaxanthin	Food additive E161e (not approved in the EU and USA) Anti-inflammatory effects in macrophages	[74,82]
Microalgae Haloarchaea Bacteria	Phytoene	Antitumoral activity	[8,78,83]
Microalgae Bacteria	Lycopene	Reduction risk of atherosclerosis and coronary heart disease Antioxidant activity Antiulcer activity Gene regulation Gap-junction communication activity Immune modulation Antitumor activity	[8,84,85,86]
Microalgae Haloarchaea	Salinixanthin	Anticancer activity (human liver cancer cell lines showed dose-dependent cytotoxicity of the carotenoids)	[8]

**Table 2:** IUPAC name, molecular formula and chemical structure of the most marketed carotenoids.

Common Name	IUPAC Name	Molecular Formula	Chemical Structure	Reference
Astaxanthin	3,3′-Dihydroxy-β,β-carotene-4,4′-dione	C_40_H_52_O_4_	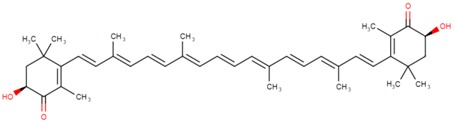 (3*S*,3′*S*)-3,3′-dihydroxy-β,β-carotene-4,4′-dione	[1]
β-Carotene	β,β-Carotene	C_40_H_56_	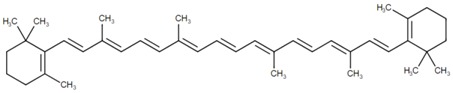 *all*-*trans*-β-carotene	[2]
Canthaxanthin	β,β-Carotene-4,4′-dione	C_40_H_52_O_2_	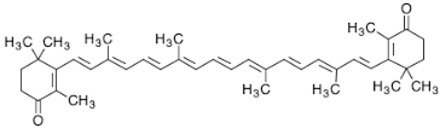 *trans-*β-carotene-4,4′-dione	[3]
β-Cryptoxanthin	β,β-Caroten-3-ol	C_40_H_56_O	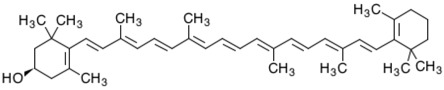 (3*R*)-β,β-Caroten-3-ol	[4]
Fucoxanthin	3,5′-Dihydroxy-8-oxo-6′,7′-didehydro-5,6-epoxy-5,6,7,8,5′,6′-hexahydro-β,β-caroten-3′-yl acetate	C_42_H_58_O_6_	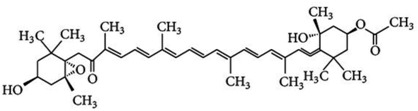 (3*S*,5*R*,6*S*,3′*S*,5′*R*,6′*R*)-3,5′-dihydroxy-8-oxo-6′,7′-didehydro-5,6-epoxy-5,6,7,8,5′,6′-hexahydro-β,β-caroten-3′-yl acetate	[5]
Lycopene	ψ,ψ-Carotene	C_40_H_56_	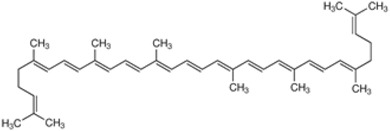 *all*-*trans*-lycopene	[6]
Lutein	β-ϵ-Carotene-3,3′-diol	C_40_H_56_O_2_	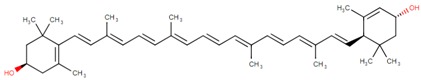 (3*R*,3′*R*,6′*R*)-β,ε-carotene-3,3′-diol	[7]
Zeaxanthin	β,β-Carotene-3,3′-diol	C_40_H_56_O_2_	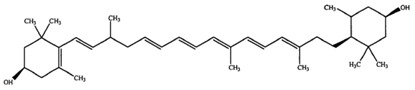 (3*R*,3′*R*)-β,β-carotene-3,3′-diol	[7]
Violaxanthin	5,5′,6,6′-Tetrahydro-5,6:5′,6′-diepoxy-β,β-carotene-3,3′-diol	C_40_H_56_O_4_	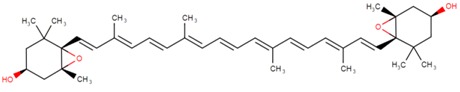 (3*S*,3′*S*,5*R*,5′*R*,6*S*,6′*S*)-5,5′,6,6′-tetrahydro-5,6:5′,6′-diepoxy-β,β-carotene-3,3′-diol	[8]

**Table 3:** IUPAC name, molecular formula and chemical structure of the rare carotenoids.

Common Name	IUPAC Name	Molecular Formula	Chemical Structure	Reference
Bacterioruberin	(2*S*,2′*S*)-2,2′-Bis(3-hydroxy-3-methylbutyl)-3,4,3′,4′-tetradehydro-1,2,1′,2′-tetrahydro-y,y-carotene-1,1′-diol	C_50_H_76_O_4_	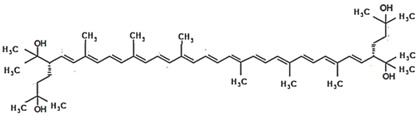	[8]
Myxol	(3*R*,3′*E*)-3′,4′-Didehydro-1′,2′-dihydro-β,ψ-carotene-1′,2′,3-triol	C_40_H_56_O_3_	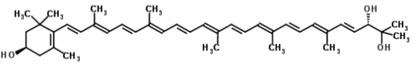	[132]
Salinixanthin	(3′*E*)-2′-Hydroxy-4-oxo-3′,4′-didehydro-1′,2′-dihydro-β,ψ-caroten-1′-yl 6-O-(13-methyltetradecanoyl)-β-D-glucopyranoside	C_61_H_92_O_9_	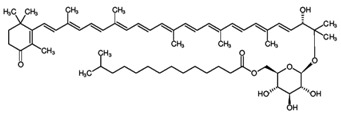	[137]
Saproxanthin	(3′*Z*)-3′,4′-Didehydro-1′,2′-dihydro-β,ψ-carotene-1′,3-diol	C_40_H_56_O_2_	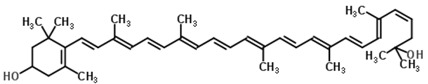	[134]
Sioxanthin	(2′*S*)-1′-(β-D-Glucopyranosyloxy)-3′,4′-didehydro-1′,2′-dihydro-Φ,Ψ-caroten-2′-ol	C_46_H_62_O_7_	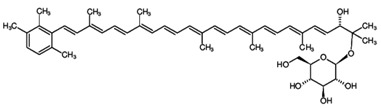	[140]
Siphonaxanthin	(3*R*,3′*R*,6′*R*)-3,3′,19-Trihydroxy-4′,5′-didehydro-5′,6′,7,8-tetrahydro-β,β-caroten-8-one	C_40_H_56_O_4_	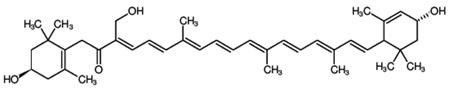	[141]

## References

[1] Nisar N., Li L., Lu S., Khin N.C., Pogson B.J. (2015). Carotenoid metabolism in plants. Mol. Plant.

[2] Zhang J., Sun Z., Sun P., Chen T., Chen F. (2014). Microalgal carotenoids: Beneficial effects and potential in human health. Food Funct..

[3] Fiedor J., Burda K. (2014). Potential role of carotenoids as antioxidants in human health and disease. Nutrients.

[4] Yatsunami R., Ando A., Yang Y., Takaichi S., Kohno M., Matsumura Y., Ikeda H., Fukui T., Nakasone K., Fujita N. (2014). Identification of carotenoids from the extremely halophilic archaeon *Haloarcula japonica*. Front. Microbiol..

[5] Goodwin T.W., Britton G., Goodwin T.W. (1980). Distribution and analysis of carotenoids. Plant Pigments.

[6] Cunningham F.X., Gantt E. (1998). Genes and enzymes of carotenoid biosynthesis in plants. Annu. Rev. Plant Physiol. Plant Mol. Biol..

[7] Mata-Gómez L.C., Montañez J.C., Méndez-Zavala A., Aguilar C.N. (2014). Biotechnological production of carotenoids by yeasts: An overview. Microb. Cell Fact..

[8] Rodrigo-Baños M., Garbayo I., Vílchez C., Bonete M.J., Martínez-Espinosa R.M. (2015). Carotenoids from Haloarchaea and their potential in biotechnology. Mar. Drugs.

[9] Gouveia L., Coutinho C., Mendonça E., Batista A.P., Sousa I., Bandarra N.M., Raymundo A. (2008). Functional biscuits with PUFA-ω3 from *Isochrysis galbana*. J. Sci. Food Agric..

[10] Vílchez C., Forján E., Cuaresma M., Bédmar F., Garbayo I., Vega J.M. (2011). Marine carotenoids: Biological functions and commercial applications. Mar. Drugs.

[11] Forján E., Vílchez Lobato C., Vega Piqueres J.M. (2014). Biotecnología de Microalgas.

[12] Othman R., Mohd Zaifuddin F.A., Hassan N.M. (2014). Carotenoid biosynthesis regulatory mechanisms in plants. J. Oleo Sci..

[13] Giuliano G. (2014). Plant carotenoids: Genomics meets multi-gene engineering. Curr. Opin. Plant Biol..

[14] Burton G.W., Foster D.O., Perly B., Slater T.F., Smith I.C., Ingold K.U. (1985). Biological antioxidants. Philos. Trans. R. Soc. Lond. B Biol. Sci..

[15] Gammone M.A., Riccioni G., D’Orazio N. (2015). Marine carotenoids against oxidative stress: Effects on human health. Mar. Drugs.

[16] LaFountain A.M., Prum R.O., Frank H.A. (2015). Diversity, physiology, and evolution of avian plumage carotenoids and the role of carotenoid-protein interactions in plumage color appearance. Arch. Biochem. Biophys..

[17] Namitha K.K., Negi P.S. (2010). Chemistry and biotechnology of carotenoids. Crit. Rev. Food Sci. Nutr..

[18] Zile M.H. (1998). Vitamin A and embryonic development: An overview. J. Nutr..

[19] Rivera S.M., Canela-Garayoa R. (2012). Analytical tools for the analysis of carotenoids in diverse materials. J. Chromatogr. A.

[20] Kaulmann A., Bohn T. (2014). Carotenoids, inflammation, and oxidative stress—Implications of cellular signaling pathways and relation to chronic disease prevention. Nutr. Res..

[21] Gupta C., Prakash D. (2014). Phytonutrients as therapeutic agents. J. Complement. Integr. Med..

[22] Ascenso A., Ribeiro H., Marques H.C., Oliveira H., Santos C., Simões S. (2014). Chemoprevention of photocarcinogenesis by lycopene. Exp. Dermatol..

[23] Woodside J.V., McGrath A.J., Lyner N., McKinley M.C. (2015). Carotenoids and health in older people. Maturitas.

[24] Freeman H.D., Valuska A.J., Taylor R.R., Ferrie G.M., Grand A.P., Leighty K.A. (2016). Plumage variation and social partner choice in the greater flamingo (*Phoenicopterus roseus*). Zoo Biol..

[25] Duarte R.C., Flores A.A.V., Stevens M. (2017). Camouflage through colour change: Mechanisms, adaptive value and ecological significance. Philos. Trans. R. Soc. Lond. B Biol. Sci..

[26] Del Campo J.A., García-González M., Guerrero M.G. (2007). Outdoor cultivation of microalgae for carotenoid production: Current state and perspectives. Appl. Microbiol. Biotechnol..

[27] Mandelli F., Miranda V.S., Rodrigues E., Mercadante A.Z. (2012). Identification of carotenoids with high antioxidant capacity produced by extremophile microorganisms. World J. Microbiol. Biotechnol..

[28] Jehlička J., Edwards H.G., Oren A. (2013). Bacterioruberin and salinixanthin carotenoids of extremely halophilic Archaea and Bacteria: A Raman spectroscopic study. Spectrochim. Acta Part A Mol. Biomol. Spectrosc..

[29] Davies J., Ryan K.S. (2012). Introducing the parvome: Bioactive compounds in the microbial world. ACS Chem. Biol..

[30] Gupta S., Abu-Ghannam N. (2011). Bioactive potential and possible health effects of edible brown seaweeds. Trends Food Sci. Technol..

[31] Stengel D.B., Connan S., Popper Z.A. (2011). Algal chemodiversity and bioactivity: Sources of natural variability and implications for commercial application. Biotechnol. Adv..

[32] Research and Innovation Website of the European Commission. http://ec.europa.eu/research.

[33] European Network to Advance Carotenoid Research and Applications in Agro-Food and Health. http://www.cost.eu/COST_Actions/ca/CA15136.

[34] Spanish Network of Carotenoids. http://www.ictan.csic.es/1693/cared-red-espanola-de-carotenoides-desde-los-microbios-y-plantas-a-los-alimentos-y-la-salud/.

[35] International Carotenoid Society. http://www.carotenoidsociety.org.

[36] Ibero-American Program of Science and Technology for Development—Carotenoids in Agro-Food and Health Section. http://www.cyted.org/es/ibercarot.

[37] Zhang L., An R., Wang J., Sun N., Zhang S., Hu J., Kuai J. (2005). Exploring novel bioactive compounds from marine microbes. Curr. Opin. Microbiol..

[38] Bhatnagar I., Kim S.K. (2010). Immense essence of excellence: Marine microbial bioactive compounds. Mar. Drugs.

[39] Habbu P., Warad V., Shastri R., Madagundi S., Kulkarni V.H. (2016). Antimicrobial metabolites from marine microorganisms. Chin. J. Nat. Med..

[40] Felczykowska A., Bloch S.K., Nejman-Faleńczyk B., Barańska S. (2012). Metagenomic approach in the investigation of new bioactive compounds in the marine environment. Acta Biochim. Pol..

[41] Gurgui C., Piel J. (2010). Metagenomic approaches to identify and isolate bioactive natural products from microbiota of marine sponges. Methods Mol. Biol..

[42] Schweder T., Lindequist U., Lalk M. (2005). Screening for new metabolites from marine microorganisms. Adv. Biochem. Eng. Biotechnol..

[43] Markou G., Nerantzis E. (2013). Microalgae for high-value compounds and biofuels production: A review with focus on cultivation under stress conditions. Biotechnol. Adv..

[44] Calegari-Santos R., Diogo R.A., Fontana J.D., Bonfim T.M. (2016). Carotenoid production by halophilic Archaea under different culture conditions. Curr. Microbiol..

[45] García-González M., Moreno J., Manzano J.C., Florencio F.J., Guerrero M.G. (2005). Production of *Dunaliella salina* biomass rich in 9-cis-β-carotene and lutein in a closed tubular photobioreactor. J. Biotechnol..

[46] Misawa N. (2011). Carotenoid β-ring hydroxylase and ketolase from marine bacteria- promiscuous enzymes for synthesizing functional xantophylls. Mar. Drugs.

[47] Marshall C.P., Leuko S., Coyle C.M., Walter M.R., Burns B.P., Neilan B.A. (2007). Carotenoid analysis of halophilic archaea by resonance Raman spectroscopy. Astrobiology.

[48] Relevy N.Z., Harats D., Harari A., Ben-Amotz A., Bitzur R., Rühl R., Shaish A. (2015). Vitamin A-deficient diet accelerated atherogenesis in apolipoprotein E(-/-) Mice and dietary β-carotene prevents this consequence. Biomed Res. Int..

[49] Sluijs I., Cadier E., Beulens J.W., van der A D.L., Spijkerman A.M., van der Schouw Y.T. (2015). Dietary intake of carotenoids and risk of type 2 diabetes. Nutr. Metab. Cadrivasc. Dis..

[50] Boeke C.E., Tamimi R.M., Berkey C.S., Colditz G.A., Eliassen A.H., Malspeis S., Willett W.C., Frazier A.L. (2014). Adolescent carotenoid intake and benign breast disease. Pediatrics.

[51] Takaichi S. (2011). Carotenoids in algae: Distributions, biosynthesis and functions. Mar. Drugs.

[52] Yokoyama A., Izumida H., Miki W. (1994). Production of astaxanthin and 4-ketozeaxanthin by the marine bacterium *Agrobacterium aurantiacum*. Biosci. Biotechnol. Biochem..

[53] Dhankar J., Kadian S.S., Sharma A. (2012). Astaxanthin: A potential carotenoid. Int. J. Pharm. Sci. Res..

[54] Fassett R.G., Coombes J.S. (2012). Astaxanthin in cardiovascular health and disease. Molecules.

[55] Higuera-Ciapara I., Félix-Valenzuela L., Goycoolea F.M. (2006). Astaxanthin: A review of its chemistry and applications. Crit. Rev. Food Sci. Nutr..

[56] Riccioni G., D’Orazio N., Franceschelli S., Speranza L. (2011). Marine carotenoids and cardiovascular risk markers. Mar. Drugs.

[57] Tanaka T., Shnimizu M., Moriwaki H. (2012). Cancer chemoprevention by carotenoids. Molecules.

[58] Peng J., Yuan J.P., Wu C.F., Wang J.H. (2011). Fucoxanthin, a marine carotenoid present in brown seaweeds and diatoms: Metabolism and bioactivities relevant to human health. Mar. Drugs.

[59] Afolayan A.F., Bolton J.J., Lategan C.A., Smith P.J., Beukes D.R. (2008). Fucoxanthin, tetraprenylatedtoluquinone and toluhydroquinone metabolites from *Sargassum heterophyllum* inhibit the in vitro growth of the malaria parasite *Plasmodium falciparum*. Z. Naturforsch. C.

[60] Woo M.N., Jeon S.M., Shin Y.C., Lee M.K., Kang M.A., Choi M.S. (2009). Anti-obese property of fucoxanthin is partly mediated by altering lipid-regulating enzymes and uncoupling proteins of visceral adipose tissue in mice. Mol. Nutr. Food Res..

[61] Shaina M., Hameed A., Lin S.Y., Lee R.J., Lee M.R., Young C.C. (2014). *Gramella planctonica* sp. Nov., a zeaxanthin-producing bacterium isolated from surface seawater and emended descriptions of *Gramella estuarii* and *Gramella echinicola*. Antonie Leeuwenhoek.

[62] Huang Y.M., Dou H.L., Huang F.F., Xu X.R., Zou Z.Y., Lin X.M. (2015). Effect of supplemental lutein and zeaxanthin on serum, macular pigmentation, and visual performance in patients with early age-related macular degeneration. Biomed Res. Int..

[63] Bernstein P.S., Li B., Vachali P.P., Gorusupudi A., Shyam R., Henriksen B.S., Nolan J.M. (2016). Lutein, zeaxanthin, and meso-zeaxanthin: The basic and clinical science underlying carotenoid-based nutritional interventions against ocular disease. Prog. Retinal Eye Res..

[64] Lu M.S., Fang Y.J., Chen Y.M., Luo W.P., Pan Z.Z., Zhong X., Zhang C.X. (2015). Higher intake of carotenoid is associated with a lower risk of colorectal cancer in Chinese adults: A case-control study. Eur. J. Nutr..

[65] Wang L., Li B., Pan M.X., Mo X.F., Chen Y.M., Zhang C.X. (2014). Specific carotenoid intake is inversely associated with the risk of breast cancer among Chinese women. Br. J. Nutr..

[66] Ghodratizadeh S., Kanbak G., Beyramzadeh M., Dikmen Z.G., Memarzadeh S., Habibian R. (2014). Effect of carotenoid β-cryptoxanthin on cellular and humoral immune response in rabbit. Vet. Res. Commun..

[67] Granado-Lorencio F., de Las Heras L., Millán C.S., García-López F.J., Blanco-Navarro I., Pérez-Sacristán B., Domínguez G. (2014). β-Cryptoxanthin modulates the response to phytosterols in post-menopausal women carrying NPC1L1 L272L and ABCG8 A632 V polymorphisms: An exploratory study. Genes Nutr..

[68] Chisté R.C., Freitas M., Mercadante A.Z., Fernandes E. (2014). Carotenoids are effective inhibitors of in vitro hemolysis of human erythrocytes, as determined by a practical and optimized cellular antioxidant assay. J. Food Sci..

[69] Min K.B., Min J.Y. (2016). Association between leukocyte telomere length and serum carotenoid in US adults. Eur. J. Nutr..

[70] Liu X.R., Wang Y.Y., Dan X.G., Kumar A., Ye T.Z., Yu Y.Y., Yang L.G. (2015). Anti-inflammatory potential of β-cryptoxanthin against LPS-induced inflammation in mouse Sertoli cells. Reprod. Toxicol..

[71] Ozaki K., Okamoto M., Fukasawa K., Iezaki T., Onishi Y., Yoneda Y., Sugiura M., Hinoi E. (2015). Daily intake of β-cryptoxanthin prevents bone loss by preferential disturbance of osteoclastic activation in ovariectomized mice. J. Pharmacol. Sci..

[72] Aasen A.J., Liaaen-Jensen S. (1996). The carotenoids of flexibacteria: II. A new xanthophyll from Saprospira grandis. Acta Chem. Scand..

[73] Francis G.W., Hertzberg S., Andersen K., Liaaen-Jensen S. (1970). New carotenoid glycosides from *Oscillatoria limosa*. Phytochemistry.

[74] Raposo M.F., De Morais A.M., De Morais R.M. (2015). Carotenoids from marine microalgae: A valuable natural source for the prevention of chronic diseases. Mar. Drugs.

[75] Sulich A., Hamułka J., Nogal D. (2015). Dietary sources of lutein in adults suffering eye disease (AMD/cataracts). Rocz. Państwowego Zakl. Hig..

[76] Nataraj J., Manivasagam T., Thenmozhi A.J., Essa M.M. (2016). Lutein protects dopaminergic neurons against MPTP-induced apoptotic death and motor dysfunction by ameliorating mitochondrial disruption and oxidative stress. Nutr. Neurosci..

[77] Han H., Cui W., Wang L., Xiong Y., Liu L., Sun X., Hao L. (2015). Lutein prevents high fat diet-induced atherosclerosis in ApoE-deficient mice by inhibiting NADPH oxidase and increasing PPAR expression. Lipids.

[78] Mathews-Roth M.M. (1982). Antitumor activity of β-carotene, canthaxanthin and phytoene. Oncology.

[79] Mordi R.C., Walton J.C. (2016). Identification of products from canthaxanthin oxidation. Food Chem..

[80] Surai P.F. (2012). The antioxidant properties of canthaxanthin and its potential effects in the poultry eggs and on embryonic development of the chick, Part 1. World’s Poult. Sci. J..

[81] Kent M., Welladsen H.M., Mangott A., Li Y. (2015). Nutritional evaluation of Australian microalgae as potential human health supplements. PLoS ONE.

[82] Soontornchaiboon W., Joo S.S., Kim S.M. (2012). Anti-inflammatory effects of violaxanthin isolated from microalga *Chlorella ellipsoidea* in RAW 264.7 macrophages. Biol. Pharm. Bull..

[83] Issouf M., Mearns S.A., Fraser K., Hodgson R. (2006). Biological Production of Zeaxanthin and Carotenoid Biosynthesis Control. World Intellectual Property Organization (WIPO) Patent Application.

[84] Viuda-Martos M., Sánchez-Zapata E., Sayas-Barberá E., Sendra E., Pérez-Álvarez J.A., Fernández-López J. (2014). Tomato and tomato byproducts. Human health benefits of lycopene and its application to meat products: A review. Crit. Rev. Food Sci. Nutr..

[85] Rao A.V., Rao L.G. (2007). Carotenoids and human health. Pharmacol. Res..

[86] Igielska-Kalwat J., Gościańska J., Nowak I. (2015). Carotenoids as natural antioxidants. Postepy Hig. Med. Dosw. (Online).

[87] Ambati R.R., Phang S.M., Ravi S., Aswathanarayana R.G. (2014). Astaxanthin: Sources, extraction, stability, biological activities and its commercial applications—A review. Mar. Drugs.

[88] Schmidt I., Schewe H., Gassel S., Jin C., Buckingham J., Hümbelin M., Sandmann G., Schrader J. (2011). Biotechnological production of astaxanthin with *Phaffia rhodozyma*/*Xanthophyllomyces dendrorhous*. Appl. Microbiol. Biotechnol..

[89] Mogedas B., Casal C., Forján E., Vílchez C. (2009). Beta-carotene production enhancement by UV-A radiation in *Dunaliella bardawil* cultivated in laboratory reactors. J. Biosci. Bioeng..

[90] Milne G.W.A. (2005). Gardner’s Commercially Important Chemicals: Synonyms, Trade Names, and Properties.

[91] Stutz H., Bresgen N., Eckl P.M. (2015). Analytical tools for the analysis of β-carotene and its degradation products. Free Radic. Res..

[92] Englert M., Hammann S., Vetter W. (2015). Isolation of β-carotene, α-carotene and lutein from carrots by countercurrent chromatography with the solvent system modifier benzotrifluoride. J. Chromatogr. A.

[93] Tanvetyanon T., Bepler G. (2008). Beta-carotene in multivitamins and the possible risk of lung cancer among smokers versus former smokers: A meta-analysis and evaluation of national brands. Cancer.

[94] Brulc L., Simonovska B., Vovk I., Glavnik V. (2013). Determination of egg yolk xanthophylls by isocratic high-performance liquid chromatography. J. Chromatogr. A.

[95] Kathiresan S., Chandrashekar A., Ravishankar G.A., Sarada R. (2015). Regulation of astaxanthin and its intermediates through cloning and genetic transformation of β-carotene ketolase in *Haematococcus pluvialis*. J. Biotechnol.

[96] Rostami F., Razavi S.H., Sepahi A.A., Gharibzahedi S.M. (2014). Canthaxanthin biosynthesis by *Dietziana tronolimnaea* HS-1: Effects of inoculation and aeration rate. Braz. J. Microbiol..

[97] Hojjati M., Razavi S.H., Rezaei K., Gilani K. (2014). Stabilization of canthaxanthin produced by *Dietziana tronolimnaea* HS-1 with spray drying microencapsulation. J. Food Sci. Technol..

[98] Heying E.K., Tanumihardjo J.P., Vasic V., Cook M., Palacios-Rojas N., Tanumihardjo S.A. (2014). Biofortified orange maize enhances β-cryptoxanthin concentrations in egg yolks of laying hens better than tangerine peel fortificant. J. Agric. Food Chem..

[99] Wei X., Chen C., Yu Q., Gady A., Yu Y., Liang G., Gmitter F.G. (2014). Comparison of carotenoid accumulation and biosynthetic gene expression between Valencia and Rohde Red Valencia sweet oranges. Plant Sci..

[100] Burri B.J. (2015). Beta-cryptoxanthin as a source of vitamin A. J. Sci. Food Agric..

[101] Li D., Xiao Y., Zhang Z., Liu C. (2015). Light-induced oxidation and isomerization of all-trans-β-cryptoxanthin in a model system. J. Photochem. Photobiol. B Biol..

[102] Burri B.J., La Frano M.R., Zhu C. (2016). Absorption, metabolism, and functions of β-cryptoxanthin. Nutr. Rev..

[103] Kim S.M., Jung Y.J., Kwon O.N., Cha K.H., Um B.H., Chung D., Pan C.H. (2012). A potential commercial source of fucoxanthin extracted from the microalga *Phaeodactylum tricornutum*. Appl. Biochem. Biotechnol..

[104] Crupi P., Toci A.T., Mangini S., Wrubl F., Rodolfi L., Tredici M.R., Coletta A., Antonacci D. (2013). Determination of fucoxanthin isomers in microalgae (*Isochrysis* sp.) by high-performance liquid chromatography coupled with diode-array detector multistage mass spectrometry coupled with positive electrospray ionization. Rapid Commun. Mass Spectrom..

[105] Martin L.J. (2015). Fucoxanthin and its metabolite fucoxanthinol in cancer prevention and treatment. Mar. Drugs.

[106] Satomi Y. (2017). Antitumor and Cancer-preventative Function of Fucoxanthin: A Marine Carotenoid. Anticancer Res..

[107] Naziri D., Hamidi M., Hassanzadeh S., Tarhriz V., Maleki Zanjani B., Nazemyieh H., Hejazi M.A., Hejazi M.S. (2014). Analysis of carotenoid production by *Halorubrum* sp. TBZ126; an extremely halophilic archeon from Urmia Lake. Adv. Pharm. Bull..

[108] Cuaresma M., Casal C., Forján E., Vílchez C. (2011). Productivity and selective accumulation of carotenoids of the novel extremophile microalga *Chlamydomonas acidophila* grown with different carbon sources in batch systems. J. Ind. Microbiol. Biotechnol..

[109] Altemimi A., Lightfoot D.A., Kinsel M., Watson D.G. (2015). Employing response surface methodology for the optimization of ultrasound assisted extraction of lutein and β-carotene from spinach. Molecules.

[110] Prabhu S., Rekha P.D., Arun A.B. (2014). Zeaxanthin biosynthesis by members of the genus *Muricauda*. Pol. J. Microbiol..

[111] Costa S., Giannantonio C., Romagnoli C., Barone G., Gervasoni J., Perri A., Zecca E. (2015). Lutein and zeaxanthin concentrations in formula and human milk samples from italian mothers. Eur. J. Clin. Nutr..

[112] Li X.R., Tian G.Q., Shen H.J., Liu J.Z. (2015). Metabolic engineering of *Escherichia coli* to produce zeaxanthin. J. Ind. Microbiol. Biotechnol..

[113] Yamamoto H.Y., Chang J.L., Aihara M.S. (1967). Light-induced interconversion of violaxanthin and zeaxanthin in New Zealand spinach-leaf segments. Biochim. Biophys. Acta Gen. Subj..

[114] Yamamoto H.Y., Kamite L., Wang Y.Y. (1972). An ascorbate-induced absorbance change in chloroplasts from violaxanthin de-epoxidation. Plant Physiol..

[115] Sapozhnikov D.I. (1973). Investigation on the violaxanthin cycle. Pure Appl. Chem..

[116] Pfündel E., Strasser R.J. (1988). Violaxanthin de-epoxidase in etiolated leaves. Photosynth. Res..

[117] Winter K., Königer M. (1989). Dithiothreitol, an inhibitor of violaxanthin de-epoxidation, increases the susceptibility of leaves of *Nerium oleander* L. to photoinhibition of photosynthesis. Planta.

[118] Hallin E.I., Guo K., Åkerlund H.E. (2015). Violaxanthin de-epoxidase disulphides and their role in activity and thermal stability. Photosynth. Res..

[119] Demmig-Adams B., Winter K., Krüger A., Czygan F.C. (1989). Zeaxanthin and the induction and relaxation kinetics of the dissipation of excess excitation energy in leaves in 2% O_2_, 0% CO_2_. Plant Physiol..

[120] Li Y., Walton D.C. (1990). Violaxanthin is an abscisic acid precursor in water-stressed dark-grown bean leaves. Plant Physiol..

[121] Neuman H., Galpaz N., Cunningham F.X., Zamir D., Hirschberg J. (2014). The tomato mutation nxd1 reveals a gene necessary for neoxanthin biosynthesis and demonstrates that violaxanthin is a sufficient precursor for abscisic acid biosynthesis. Plant J..

[122] Kang B., Yoon H.-S. (2015). The application of two-step linear temperature program to thermal analysis for monitoring the lipid induction of *Nostoc* sp. KNUA003 in large scale cultivation. Enzyme Microb. Technol..

[123] Bhati R., Mallick N. (2016). Carbon dioxide and poultry waste utilization for production of polyhydroxyalkanoate biopolymers by *Nostoc muscorum* Agardh: A sustainable approach. J. Appl. Phycol..

[124] Mona S., Kaushik A., Kaushik C.P. (2013). Prolonged hydrogen production by *Nostoc* in photobioreactor and multi-stage use of the biological waste for column biosorption of some dyes and metals. Biomass Bioenergy.

[125] Singh S., Verma E., Niveshika, Tiwari B., Mishra A.K. (2016). Exopolysaccharide production in *Anabaena* sp. PCC 7120 under different CaCl_2_ regimes. Physiol. Mol. Biol. Plants.

[126] Ten L.N., Chae S.M., Yoo S.A. (2015). Production of poly-3-hydroxybutyrate by cyanobacterium *Anabaena sp*. BD47. Chem. Nat. Compd..

[127] Shindo K., Misawa N. (2014). New and rare carotenoids isolated from marine bacteria and their antioxidant activities. Mar. Drugs.

[128] Strand A., Shivaji S., Liaaen-Jensen S. (1997). Bacterial carotenoids 55. C_50_-carotenoids 25. Revised structures of carotenoids associated with membranes in psychrotrophic *Micrococcus roseus*. Biochem. Syst. Ecol..

[129] Shahmohammadi H.R., Asgarani E., Terato H., Saito T., Ohyama Y., Gekko K., Yamamoto O., Ide H. (1998). Protective roles of bacterioruberin and intracellular KCl in the resistance of *Halobacterium salinarium* against DNA-damaging agents. J. Radiat. Res..

[130] Kelly M., Norgard S., Liaaen-Jensen S. (1970). Bacterial carotenoids. 31. C50-carotenoids 5. Carotenoids of *Halobacterium salinarium*, especially bacterioruberin. Acta Chem. Scand..

[131] Takaichi S., Mochimaru M., Maoka T. (2006). Presence of free myxol and 4-hydroxymyxol and absence of myxol glycosides in *Anabaena variabilis* ATCC 29413, and proposal of a biosynthetic pathway of carotenoids. Plant Cell Physiol..

[132] Manh H.D., Matsuo Y., Katsuta A., Matsuda S., Shizuri Y., Kasai H. (2008). *Robiginitalea myxolifaciens* sp. nov., a novel myxol-producing bacterium isolated from marine sediment, and emended description of the genus *Robiginitalea*. Int. J. Syst. Evol. Microbiol..

[133] Arima H., Horiguchi N., Takaichi S., Kofuji R., Ishida K.I., Wada K., Sakamoto T. (2012). Molecular genetic and chemotaxonomic characterization of the terrestrial cyanobacterium *Nostoc commune* and its neighboring species. FEMS Microbiol. Ecol..

[134] Shindo K., Kikuta K., Suzuki A., Katsuta A., Kasai H., Yasumoto-Hirose M., Takaichi S. (2007). Rare carotenoids, (3R)-saproxanthin and (3R, 2′ S)-myxol, isolated from novel marine bacteria (*Flavobacteriaceae*) and their antioxidative activities. Appl. Microbiol. Biotechnol..

[135] De Lourdes Moreno M., Sánchez-Porro C., García M.T., Mellado E. (2012). Carotenoids’ production from halophilic bacteria. Methods Mol. Biol..

[136] Lutnaes B.F., Oren A., Liaaen-Jensen S. (2002). New C(40)-carotenoid acyl glycoside as principal carotenoid in *Salinibacter ruber*, an extremely halophilic eubacterium. J. Nat. Prod..

[137] Balashov S.P., Imasheva E.S., Lanyi J.K. (2006). Induced chirality of the light-harvesting carotenoid salinixanthin and its interaction with the retinal of xanthorhodopsin. Biochemistry.

[138] Sakata T., Yasumoto H. (1991). Colony formation by algicidal *Saprospira* sp. on marine agar plates. Nippon Suisan Gakkaishi.

[139] Takatani N., Nishida K., Sawabe T., Maoka T., Miyashita K., Hosokawa M. (2014). Identification of a novel carotenoid, 2′-isopentenylsaproxanthin, by *Jejuia pallidilutea* strain 11shimoA1 and its increased production under alkaline condition. Appl. Microbiol. Biotechnol..

[140] Richter T.K., Hughes C.C., Moore B.S. (2015). Sioxanthin, a novel glycosylated carotenoid, reveals an unusual subclustered biosynthetic pathway. Environ. Microbiol..

[141] Walton T.J., Britton G., Goodwin T.W., Diner B., Moshier S. (1970). The structure of siphonaxanthin. Phytochemistry.

[142] Kageyamam A., Yokohama Y., Shimura S., Ikawa T. (1977). An efficient excitation energy transfer from a carotenoid, siphonaxanthin to chlorophyll a observed in a deep-water species of chlorophycean seaweed. Plant Cell Physiol..

[143] Yokohama Y., Kageyama A., Ikawa T., Shimura S. (1977). A carotenoid characteristic of chlorophycean seaweeds living in deep coastal waters. Bot. Mar..

[144] Sugawara T., Ganesan P., Li Z., Manabe Y., Hirata T. (2014). Siphonaxanthin, a green algal carotenoid, as a novel functional compound. Mar. Drugs.

[145] Hendrickson S.J., Willett W.C., Rosner B.A., Eliassen A.H. (2013). Food predictors of plasma carotenoids. Nutrients.

[146] EFSA (European Food Safety Authority) (2008). Scientific Opinion of the Panel on Dietetic Products, Nutrition and Allergies on a request from the EC on Food-Based Dietary Guidelines. EFSA J..

[147] Freitas A.C., Rodrigues D., Rocha-Santos T.A., Gomes A.M., Duarte A.C. (2012). Marine biotechnology advances towards applications in new functional foods. Biotechnol. Adv..

[148] El-Sohaimy S.A. (2012). Functional foods and nutraceuticals-modern approach to food science. World Appl. Sci. J..

[149] FAO: Food and Agriculture Organization of the United Nations Report on Functional Foods, Food Quality and Standards Service (AGNS). http://www.fao.org/ag/agn/agns/files/Functional_Foods_Report_Nov2007.pdf.

[150] Brower V. (1998). Nutraceuticals: Poised for a healthy slice of the healthcare market?. Nat. Biotechnol..

[151] Trottier G., Bostrom P.J., Lawrentschuk N., Fleshner N.E. (2010). Nutraceuticals and prostate cancer prevention: A current review. Nat. Rev. Urol..

[152] Gaffe R. (2010). The Current and Future Regulation of Dietary Supplements. http://www.richardjaffeesq.com/jaffe/dietarysupplements.asp.

[153] Boon C.S., Mc Clements D.J., Weiss J., Decker E.A. (2010). Factors influencing the chemical stability of carotenoids in foods. Crit. Rev. Food Sci. Nutr..

[154] Margină D., Ilie M., Grădinaru D., Androutsopoulos V.P., Kouretas D., Tsatsakis A.M. (2015). Natural products-friends or foes?. Toxicol. Lett..

[155] Gangakhedkar A., Somerville R., Jelleyman T. (2017). Carotenemia and hepatomegaly in an atopic child on an exclusion diet for a food allergy. Australas J. Dermatol..

[156] Safi K.H., Filbrun A.G., Nasr S.Z. (2014). Hypervitaminosis A causing hypercalcemia in cystic fibrosis. Case report and focused review. Ann. Am. Thorac. Soc..

[157] Gamal A.A. (2010). Biological importance of marine algae. Saudi Pharm. J..

[158] Bule M.H., Ahmed I., Maqbool F., Bilal M., Iqbal H.M.N. (2018). Microalgae as a source of high-value bioactive compounds. Front. Biosci. (Sch. Ed.).

[159] Kadam S.U., Prabhasankar P. (2010). Marine foods as functional ingredients in bakery and pasta products. Food Res. Int..

[160] Plaza M., Herrero M., Cifuentes A., Ibáñez E. (2009). Innovative natural functional ingredients from microalgae. J. Agric. Food Chem..

[161] Vicentini A., Liberatore L., Mastrocola D. (2016). Functional foods: Trends and development of the global market. Ital. J. Food Sci..

[162] Chacon-Lee T.L., González-Mariño G.E. (2010). Microalgae for “healthy” foods-possibilities and challenges. Compr. Rev. Food Sci. Food Saf..

[163] Milledge J.J. (2012). Microalgae-Commercial Potential for Fuel, Food and Feed. Food Sci. Technol..

[164] Enzing C., Ploeg M., Barbosa M., Sijtsma L. (2014). Microalgae-Based Products for the Food and Feed Sector: An Outlook for EUROPE.

[165] Rodríguez-Amaya D.B. (2015). Status of carotenoid analytical methods and in vitro assays for the assessment of food quality and health effects. Curr. Opin. Food Sci..

[166] Gouveia L., Batista A.P., Raymundo A., Sousa I., Empis J. (2006). *Chlorella vulgaris* and *Haematococcus pluvialis* biomass as colouring and antioxidant in food emulsions. Eur. Food Res. Technol..

[167] Gouveia L., Batista A.P., Miranda A., Empis J., Raymundo A. (2007). *Chlorella vulgaris* biomass used as colouring source in traditional butter cookies. Innov. Food Sci. Emerg. Technol..

[168] Liang S., Liu X., Chen F., Chen Z. (2004). Current microalgal health food R&D activities in China. Hydrobiologica.

[169] Gantar M., Svircev Z. (2008). Microalgae and cyanobacteria: Food for thought. J. Phycol..

[170] Wijesinghe W.A.J.P., Jeon Y.J. (2011). Biological activities and potential cosmeceutical applications of bioactive components from brown seaweeds: A review. Phytochem. Rev..

[171] Shah M.M., Liang Y., Cheng J.J., Daroch M. (2016). Astaxanthin-producing green microalga *Haematococcus pluvialis:* From single cell to high value commercial products. Front. Plant Sci..

[172] Hudson E. (2008). Trend Watch: Spirulina: Healthy, Green, Versatile. http://www.portal.euromonitor.com.simsrad.net.ocs.mq.edu.

[173] Spiralyn: Promoting Good Health. http://www.foodnavigator.com/Financial-Industry/Blue-Smarties-are-back-thanks-to-Spirulina.

[174] Pooja S. (2014). Algae used as medicine and food-a short review. J. Pharm. Sci. Res..

[175] Raja R., Hemaiswarya S., Kumar N.A., Sridhar S., Rengasamy R. (2008). A perspective on the biotechnological potential of microalgae. Crit. Rev. Microbiol..

[176] Borowitzka M.A. (2013). High-value products from microalgae, their development and commercialisation. J. Appl. Phycol..

[177] Holdt S.L., Kraan S. (2011). Bioactive compounds in seaweed: Functional food applications and legislation. J. Appl. Phycol..

[178] Sweetman J.O. (2016). Developing Food Products for Consumers with Specific Dietary Needs.

